# The utilization of nanopore targeted sequencing proves to be advantageous in the identification of infections present in deceased donors

**DOI:** 10.3389/fmicb.2023.1238666

**Published:** 2023-08-16

**Authors:** Zhiyuan Yao, Yu Liu, Liying Zhan, Tao Qiu, Guang Li, Zhongbao Chen, Xiaoyu Fang, Zhou Liu, Wei Wu, Zhaomin Liao, Wenfang Xia

**Affiliations:** ^1^Department of Critical Care Medicine, Renmin Hospital of Wuhan University, Wuhan, Hubei, China; ^2^Department of Cardiology, Renmin Hospital of Wuhan University, Wuhan, Hubei, China; ^3^Cardiovascular Research Institute of Wuhan University, Wuhan, Hubei, China; ^4^Hubei Key Laboratory of Cardiology, Wuhan, Hubei, China; ^5^Department of Organ Transplantation, Renmin Hospital of Wuhan University, Wuhan, Hubei, China

**Keywords:** Chinese donation after citizen’s death, deceased donors, nanopore targeted sequencing, blood culture, third-generation sequencing

## Abstract

**Background:**

Nanopore Target Sequencing (NTS) represents a novel iteration of gene sequencing technology; however, its potential utility in the detection of infection in deceased donors has yet to be documented. The present study endeavors to assess the applicability of NTS in this domain.

**Methods:**

This retrospective study comprised a cohort of 71 patients who were under intensive care at Renmin Hospital of Wuhan University between June 2020 and January 2022. The specimens were subjected to microbiological tests utilizing NTS, culture, and other techniques, and subsequently, the diagnostic accuracy of NTS was compared with conventional methods.

**Results:**

Blood NTS exhibited a better agreement rate of 52.11% and a greater positive rate of pathogen detection than blood culture (50.70% vs. 5.63%, *p* < 0.001). In NTS of deceased donors, *Klebsiella pneumoniae*, *Escherichia coli*, and *Acinetobacter baumannii* were the most frequently found bacteria, and *Candida* was the most frequently found fungus. Blood NTS had a considerably better sensitivity for detecting clinical bloodstream infection than blood culture (62.50%: 7.14%, *p* < 0.001). These findings were supported by comparisons between blood NTS and conventional microbial detection methods (such as blood culture, glucan testing, galactomannan testing, T cell spot testing for tuberculosis infection, smear, etc.).

**Conclusion:**

The pathogen detection technology NTS has a high sensitivity and positive rate. It can more accurately and earlier detect infection in deceased donors, which could be very important for raising the donation conversion rate.

## 1. Introduction

An important form of treatment for advanced organ failure is organ transplantation. The number of organ transplants worldwide has dramatically increased despite the effects of COVID-19. The second-largest country in the world for organ transplant surgery is now China (Home, n.d.; Shi et al., 2020). One of China’s primary sources of transplantable organs at the moment is deceased donors. In recent years, the number of organ donors has increased (Home, n.d.; Shi et al., 2020). Many variables, including the experience level of the surgeons and the age of the donors, influence the outcomes of organ transplantation. One of the main reasons why recipients of transplants develop malignant outcomes is infection after the procedure (Wang et al., 2018). According to studies, infections account for 30.9% of the causes of death among kidney transplant recipients within a year (Begaj et al., 2013). Studies have revealed that 5% of donated organs had sepsis (Wolfe et al., 2019). Anti-infective therapy is advised by the guidelines for all donors who may be infected (Wolfe et al., 2019), however many donors’ infection statuses cannot be determined in a timely manner. Additionally, infections in organ donors may make it more difficult to use transplantable organs effectively (Choi et al., 2021). Therefore, early detection of illness in organ donors may aid in increasing the rate of donation.

The gold standard for diagnosing bloodstream infection is blood culture (Allerberger and Kern, 2020), but it has drawbacks such a long waiting period, a narrow detection range, and a low detection efficiency for specific bacteria and fungi. To maintain the survival of transplantable organs, deceased donors must be screened for infection within 4–12 h and some donors must undertake organ removal operations within hours or 1–2 days. Blood culture is currently unable to match the demands, thus doctors urgently require effective and quick diagnostic technologies. The third generation of gene sequencing technology is called nanopore target sequencing (NTS). It can identify DNA sequences by the current changes brought on when DNA strands are compelled to pass through membrane-encased nanopores (Tyler et al., 2018). Small size, low cost, and portability are its benefits. NTS can identify large gene sequences (Ciuffreda et al., 2021), does not rely on polymerase chain reaction technology, and requires little effort to prepare a gene library. Endophthalmitis (Huang et al., 2021), lower respiratory tract infections (Wirth et al., 2012; Charalampous et al., 2019; Chan et al., 2020), surgical site infections (Whittle et al., 2022), new coronaviruses, and respiratory viruses (Wang et al., 2020b) have all been diagnosed using NTS. There is no information available, though, about the use of NTS to check for infection in deceased donors. Therefore, in order to assess the viability and effectiveness of NTS in quickly screening for infection in such individuals, this study retrospectively assessed the NTS test results of blood samples from deceased donors.

## 2. Materials and methods

### 2.1. Design of the study and participants

From June 2020 to January 2022, deceased donors who were being cared for in the intensive care unit (ICU) at Renmin Hospital of Wuhan University (Wuhan, China) were included in this retrospective investigation. The Renmin Hospital of Wuhan University’s Ethics Committee of Clinical Research accepted this study, which complies with the Helsinki Declaration. The family of every participant gave their signed, informed consent. The organ donation procedure used in this study complies with the rules and procedures used when Chinese citizens pass away (Huang et al., 2015). Patients who did not have blood samples subjected to NTS detection met the exclusion criteria.

### 2.2. Data collection

Clinical information was retrieved from the examined medical records of deceased donors, including demographic information, medical history, results of laboratory tests, and a treatment plan. To maintain secrecy, each participant uses a predetermined number.

### 2.3. Sample collection

Peripheral venous blood samples: Within 24 h after being admitted to the intensive care unit, clinical nurses with experience took peripheral venous blood samples under stringent aseptic guidelines. (1) Collection of blood culture samples: Each time, 2 sets of blood cultures were obtained, each set from a separate puncture location. Each set contained 8–10 mL of blood that was injected into both aerobic and anaerobic bottles. (2) Collection of the NTS sample: Blood samples of at least 2 mL were taken and preserved in a specialized container.

### 2.4. Culture

After collection, peripheral venous blood samples were sent right away for culture and NTS. Using the BACTEC 9120 culture system (BD Diagnostics, Sparks, MD), the specimens were initially inoculated on Columbia blood agar basal medium (bacteria) and Sabouraud’s glucose agar medium (fungi). The isolated fungi or/and bacteria in the positive instances were identified using the MALDI Biotyper mass spectrometer (Bruker, Madison, WI) and the Vitek 2 Compact automatic identification system (BioMérieux, Marcy l’Etoile, 106 France).

### 2.5. Nanopore targeted sequencing

NTS was carried out utilizing Wang et al.’s method (Wang et al., 2020a). The QIAamp UCP Pathogen Mini Kit (Qiagen, Venlo, Netherlands) was used to extract DNA. The same sample’s 16 s rRNA, ITS1/2, and rpoB genes were amplified, and the combined barcode products were mixed at a mass ratio of 10:3:1. The 1D Lliging Kit (SQK-LSK109; Oxford Nanopore Technologies, Oxford, UK) was used to mix the combined products of the several samples in equal parts. The Min ION or Grid ION system (Oxford Nanopore Technologies, Oxford, UK) was used to sequence the library. Each batch included TE buffer measurement as a negative control. If any set threshold is met after bioinformatics analysis of the material, positive identification of bacteria or fungi is carried out.

### 2.6. The principles for identifying microbial infections and contamination

According to clinical guidelines and previous studies, pathogenic microorganisms were divided into: (1) Absolute pathogenic bacteria, such as *Salmonella* spp., *Mycobacterium tuberculosis*, *Corynebacterium diphtheriae*, etc. (2) Common opportunistic pathogens, such as *Staphylococcus aureus*, *Enterococcus* spp., *Escherichia coli*, *Pseudomonas* spp., *Klebsiella* spp., *Acinetobacter* spp. and *Candida* spp. (3) Common contaminated bacteria: such as Coagulase negative *Staphylococcus*, *Corynebacterium* spp., *Propionibacterium* spp., *Streptococcus viridans*, *Aeromonas* spp., *Bacillus* spp., and *Micrococcus* spp., etc. (Horan et al., 2008; Byrd et al., 2018; Timsit et al., 2020; Sommerstein et al., 2021; Gouel-Cheron et al., 2022).

For absolute pathogenic bacteria and common opportunistic pathogens, when one or more blood cultures were positive and NTS was positive, the pathogen was considered to be causative pathogens. For common contaminated bacteria, (1) When two or more blood cultures are positive and have infection-related symptoms (such as fever, chills, and hypotension, etc.), the pathogen is considered to be a causative pathogens; (2) When NTS is positive, the patient has infection-related symptoms and laboratory test results and cannot be explained by other reasons, and the antibiotic treatment is effective, the pathogen is considered to be a causative pathogens (Horan et al., 2008).

### 2.7. Statistical analysis

The mean and standard deviation (SD) were used to characterize data with normal distribution, the median (interquartile range, IQR), and the count (%), for non-normal distribution. For difference analysis, the T test, variance analysis, non-parametric test, and chi-square test were utilized. The effectiveness of NTS and blood culture as diagnostic tools was compared using the McNemar chi-square test. The 95% confidence interval (95% CI) and the sensitivity, specificity, positive predictive value (PPV), and negative predictive value (NPV) were provided. Statistics were judged significant at *p <* 0.05. Excel 2019 was used to gather the data, and IBM SPSS Statistics 26 Windows (Armonk, NY, USA) was utilized for the statistical analysis.

## 3. Results

### 3.1. Clinical and demographic characteristics

We looked at the medical files of 75 deceased donors. 71 patients were eventually included after four patients who refused NTS were eliminated. Table 1 displays the demographic and medical traits of the patients. The median age was 55 (46,62) years, the median length of stay in the ICU was 4 (2,5) days, and the median acute physiology and chronic health evaluation II (APACHE II) score was 20 (16,23). There were 64 men and 7 women in the study. Cerebral hemorrhage (70.42%) was the most prevalent underlying condition, followed by lung infection (61.97%) and hypertension (54.93%). When the patient entered the ICU, the white blood cell (WBC), neutrophil ratio, C-reactive protein (CRP), and procalcitonin (PCT) levels were all greater than the standard reference value. Following ICU admission, all patients received empirical anti-infective therapy, with meropenem, teicoplanin, and voriconazole being the most frequently prescribed antibiotics.

**Table 1:** Clinical and demographic characteristics of deceased donors.

Items	Deceased donors
Sex (Male)	64 (90.14%)
Age (years)	55 (46, 62)
Days in ICU	4 (2, 5)
Acute physiology and chronic health evaluation II	20 (16, 23)
Primary disease	
Cerebral hemorrhage	50 (70.42%)
Pulmonary infection	44 (61.97%)
Hypertensive	39 (54.93%)
Mechanical ventilation	71 (100.00%)
Extracorporeal membrane oxygenation	7 (9.86%)
White blood cell (×10^9^/L)	12.06 (9.58, 15.86)
Neutrophils (%)	83.21 **±** 8.51
C-reactive protein (mg/L)	111.84 **±** 71.42
Procalcitonin (ng/ml)	0.92 (0.15, 2.94)
Antibiotic	
Teicoplanin	70 (98.59%)
Meropenem	64 (90.14%)
Polymyxin B sulfate	22 (30.99%)
Imipenem and Cilastatin sodium	14 (19.72%)
Antifungal	
Voriconazole	65 (91.55%)
Micafungin Sodium	13 (18.31%)

Data are expressed as $\bar{x}$ ± SD, M (IQR) or *n* (%).

### 3.2. Pathogen detection in blood using NTS and blood culture comparison

**Figure 1:**
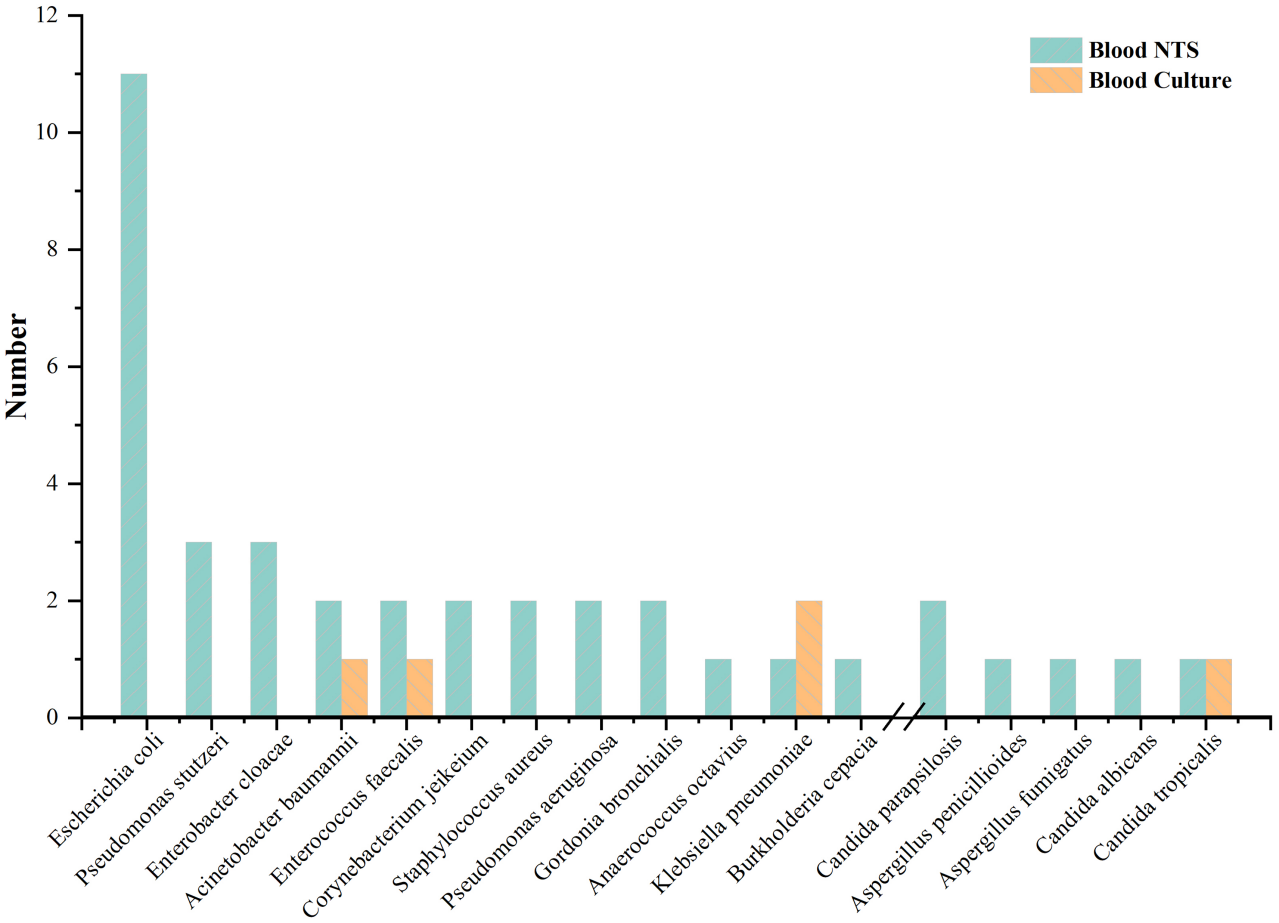
Blood nanopore target sequencing (NTS) and blood culture pathogen identification.

Blood cultures from four patients (5.63%) were positive. In blood culture, three bacteria (*Klebsiella pneumoniae*, *Enterococcus faecalis*, and *Acinetobacter baumannii*) and one fungus (*Candida tropicalis*) were found. 36 patients (50.70%) showed positive blood NTS; 21 (58.33%) had just one pathogen, and 15 (41.67%) had two or more. Blood culture was unable to detect four individuals who had bacterial and fungal co-infections according to NTS. 32 (86.49%) bacteria and 5 (13.51%) fungus at the species level were found in blood NTS. Gram-negative bacteria made up 62.50% of the identified bacteria, with *Escherichia coli* accounting for the majority (11 instances), followed by *Pseudomonas stutzeri* and *Enterobacter cloacae* (Figure 1). Supplementary Table S1 provides specific information on pathogens identified by blood culture, blood NTS, and other microbial detection techniques.

### 3.3. Blood culture and blood NTS consistency comparison

We begin by contrasting the agreement rate between blood NTS and blood culture (Table 2), as blood culture is now the gold standard for the diagnosis of bloodstream infections (Allerberger and Kern, 2020). The agreement rate was 52.11 percent, and blood NTS had a positive rate that was substantially higher than blood culture (50.70 percent vs. 5.63 percent, *p* < 0.001). According to the findings, there is a strong correlation between NTS and blood cultures, and when NTS is negative, blood cultures are more likely to turn out negatively.

**Table 2:** Blood nanopore target sequencing (NTS) and blood culture agreement rate.

		Blood culture		Sensitivity% (95%CI)	Specificity% (95%CI)	PPV% (95%CI)	NPV% (95%CI)
		+	−				
Blood NTS	+	3	33	75.00 (21.94, 98.68)	50.75(38.36, 63.05)	8.33 (2.17, 23.59)	97.14(83.38, 99.85)
	−	1	34				

PPV, Positive Predictive Value; NPV, Negative Predictive Value.

### 3.4. Comparison of blood NTS and blood culture’s diagnostic effectiveness in individuals who are infected and those who are not

After being admitted to the intensive care unit, 71 deceased donors received broad-spectrum antibiotic treatment to avoid infection. In the infection group, there were 56 patients, while in the non-infected group, there were only 15. After a thorough examination of clinical symptoms, signs (such as increased body temperature, decreased blood pressure, increased heart rate or increased respiratory rate, etc.), laboratory results (white blood cell count, neutrophil count, procalcitonin and C-reactive protein, etc.), imaging results, treatment choices, and outcomes, three skilled doctors made the infection diagnosis (Horan et al., 2008; Cui et al., 2022, 2010–2019). Only 4 of the 56 sick patients were found by blood culture; 35 were found by NTS. A blood culture revealed no false positives, however one of the 15 non-infected patients tested positive for NTS and had erroneous positives. The sensitivity of blood NTS was substantially higher than that of blood culture (62.50%: 7.14%, P<0.001) and was 55.36% higher than that of blood culture. Blood NTS’s specificity was 93.33%, while its PPV and NPV values were 97.22 and 40.00%, respectively. Blood culture’s specificity was 100%, its PPV and NPV were 100% and 22.39%, respectively (Table 3). Additionally, when we compared the outcomes of blood NTS with all other conventional methods for detecting pathogenic microorganisms (such as blood cultures, glucan tests, galactomannan tests, T cell spot tests for tuberculosis infection, smears, etc.), we discovered that blood NTS had a higher diagnostic effectiveness (see Supplementary Table S2).

**Table 3:** Blood nanopore target sequencing (NTS) and culture performance in clinical infectious disease diagnosis.

		Infection (56)	Non-infection (15)	Sensitivity% (95%CI)	Specificity% (95%CI)	PPV% (95%CI)	NPV% (95%CI)
Blood NTS	+	35	1	62.50 (48.52, 74.77)	93.33 (66.03, 99.65)	97.22 (83.80, 99.85)	40.00 (24.35, 57.79)
	−	21	14				
Blood culture	+	4	0	7.14 (2.31, 18.13)	100.00 (74.65, 100.00)	100.00 (39.58, 100.00)	22.39 (13.47, 34.52)
	−	52	15				

PPV, Positive Predictive Value; NPV, Negative Predictive Value.

### 3.5. Effect of blood NTS test results

The blood NTS of patient NO.18# revealed the presence of *Staphylococcus aureus*, while patient NO.37# revealed the presence of *Escherichia coli*, *Pseudomonas stutzeri*, and *Acinetobacter gilrohlii*. The two patients’ anti-infective regimens were modified in light of the NTS results (Table 4). The two patients’ subsequent blood culture results were negative, demonstrating that NTS can more quickly provide recommendations for therapeutic therapy.

**Table 4:** Based on the findings of nanopore target sequencing (NTS), treatment changes.

Patient ID	Putative pathogens detected by blood NTS (reads)	Changes in antibiotic
NO.18#	*Staphylococcus aureus* (96)	Teicoplanin added
NO.37#	*Escherichia coli* (572), *Pseudomonas stutzeri* (293), *Acinetobacter guillouiae* (116)	Ceftazidime upgraded to meropenem

### 3.6. The role of NTS in predicting receptor infection

In this study, we collected the pathogenic microbial test results of kidney recipients matching deceased donors in order to assess the role of NTS in predicting infection in organ transplant recipients (since the donor kidney is mainly distributed to our hospital, and other organs are distributed throughout the country, the data of the kidney recipients we obtained are the most complete.). There were 128 renal recipients in all, 86 of whom were men (87.2%) and 42 of whom were women (32.8%), with an average age of 44.98 ± 11.42 years. After surgery, 111 (86.7%) patients had blood cultures performed (positive rate: 2.7%), 121 (94.5%) patients had urine cultures performed (positive rate: 3.3%), 48 (37.5%) patients had blood NTS performed (positive rate: 77.1%), 32 (25.0%) patients had urine NTS performed (positive rate: 31.3%), and 112 (87.5%) patients had surgical site secretion cultures performed (positive rate: 1.8%). The aforementioned findings demonstrated that NTS had a considerably greater positive rate than culture (*p* < 0.001). Comparing the results of pathogenic microorganism detection between donors and recipients, we discovered that 12 recipients and their corresponding 7 donors both had the same pathogenic bacteria (all *Escherichia coli*), all of which were detected by NTS but not by culture (Table 5). As a result, NTS may be superior to culture in predicting postoperative infection in recipients. Additionally, we discovered that the diagnosis of donor-derived infection (DDI) coincided in five groups of patients (group meaning donor and its corresponding recipient) (Wolfe et al., 2019), which may suggest that NTS also has promise in screening DDI. Supplementary Table S1 of the Supplementary Material displays the complete results.

**Table 5:** The results of pathogenic microorganism examination of deceased donors and their corresponding renal recipients.

Deceased donors			Renal recipients					
Patient ID	Blood NTS results (reads)	Blood Culture results	Patient ID	Blood Culture results	Urine culture	Blood NTS results (reads)	Urine NTS results (reads)	Surgical site secretion culture results
NO.32	*Escherichia coli* (492), *Burkholderia vietnamiensis* (73)	Negative	NO.55	Negative	Negative	*Escherichia coli* (2395)	Non-implementation	Negative
			NO.56	Negative	Negative	*Escherichia coli* (5151), *Pseudomonas stutzeri* (72)	Non-implementation	*Escherichia coli*
NO.34	*Escherichia coli* (434)	Negative	NO.59	Negative	Negative	*Escherichia coli* (324), *Burkholderia cepacia* (1277), *Acinetobacter johnsonii* (4434)	Negative	Negative
			NO.60	Negative	Negative	*Escherichia coli* (89), *Streptococcus oralis* (111)	Negative	Negative
NO.36	*Pseudomonas fluorescens* (209), *Escherichia coli* (59)	Negative	NO.62	Negative	Negative	*Pseudomonas aeruginosa* (127), *Escherichia coli* (4227)	*Enterococcus gallinarum* (16)	Negative
			NO.63	Negative	Negative	*Staphylococcus aureus* (664), *Escherichia coli* (1297)，*Pseudomonas stutzeri* (228)	Negative	Negative
NO.37	*Escherichia coli* (973), *Acinetobacter guillouiae* (293), *Pseudomonas stutzeri* (116), *Corynebacterium mucifaciens* (4948)	Negative	NO.64	Negative	Negative	*Escherichia coli* (2352)	Non-implementation	Non-implementation
NO.39	*Escherichia coli* (256), *Anaerococcus octavius* (1481), *Acinetobacter haemolyticus* (218)	Negative	NO.68	Negative	Negative	*Escherichia coli* (132), *Pseudomonas luteola* (309)	Negative	Non-implementation
			NO.69	Negative	Negative	*Escherichia coli* (134), *Streptococcus oralis* (293), *Pseudomonas stutzeri* (270), *Malassezia.restricta* (361)	Negative	Negative
NO.50	*Escherichia coli* (548), *Pseudomonas hibiscicola* (203)	Negative	NO.88	non-implementation	Negative	*Pseudomonas stutzeri* (78), *Escherichia coli* (20)	Non-implementation	Negative
			NO.89	non-implementation	Negative	*Pseudomonas stutzeri* (364), *Escherichia coli* (169), *Pseudomonas oryzihabitans* (250)	Non-implementation	Negative
NO.51	*Escherichia coli* (156)	Negative	NO.90	Negative	Negative	*Escherichia coli* (67)	Non-implementation	Negative

NTS, Nanopore Target Sequencing.

## 4. Discussion

Renal artery rupture and thrombosis are just two of the harmful outcomes that can result from DDI (Wang et al., 2018; Liu et al., 2019; Tong et al., 2020), and the attributable mortality of recipients with DDI is as high as 25–33%(Benamu et al., 2017). Infection in deceased donors is not a strict prohibition against organ donation, according to earlier research (Kieslichova et al., 2019). The safe performance of liver and kidney transplantation with the prophylactic use of antibiotics is possible even if the donor has a systemic illness (even a multidrug-resistant bacterial infection) (Yuan et al., 2016), and positive transplantation outcomes are possible (Ye et al., 2017). The majority of patients, however, do not receive particular antibacterial treatment prior to organ removal surgery since the illness of deceased donors is frequently not recognized in a timely and efficient manner (Yuan et al., 2016). The majority of patients suffer from lung infections or other organ damage brought on by underlying disorders, and the majority of deceased donors have terrible underlying diseases. According to the study’s findings, cerebral hemorrhage, lung infection, and hypertension were the primary basic disorders affecting the patients. Organ removal surgery might begin at different times for deceased donors with varied circumstances. It has been challenging for doctors to maintain the organ function of such individuals because of this hazy node. Although empirical antibiotic therapy will be administered to dead donors after ICU admission, clinicians hope to get quicker, more concrete results to help them decide how to employ antimicrobial medications. Blood culture requires a lot of time and has a limited detection area, which is insufficient. Due to its broad detection range and quick detection, third-generation gene detection technology NTS has been used in a variety of industries. Its use in checking for infection in deceased donors has not, however, been documented. The effectiveness of NTS in the diagnosis of infection in such patients was therefore investigated in this study.

In this investigation, 71 individuals were included, and 37 pathogens, mostly Gram-negative bacteria (62.50%), were found in peripheral blood samples from 36 (50.70%) NTS-positive patients. A Gram-negative bacterial infection in the donor has been linked to an increased risk of DDI, according to earlier research [5]. According to this study, *Candida* was the fungus and *Klebsiella pneumoniae*, *Escherichia coli*, and *Acinetobacter baumannii* were the most prevalent infectious bacteria found in deceased donors. Pathogens that blood culture could not reliably identify, such as *Pseudomonas stutzeri*, *Enterobacter cloacae*, and *Aspergillus*, were found by blood NTS. The difference between the number of fungus found in blood NTS and blood culture—7 fungi were found in blood NTS, compared to none in blood culture—showed that NTS was more effective in detecting fungal infection and had a wider detection range. There are two sides to this NTS feature. The benefit is that it can locate suspected infectious pathogens to the greatest extent possible, particularly unique diseases that cannot be found by culture or other techniques. It may be challenging to discern between important pathogens, conditional pathogens, and normal non-pathogenic symbiotic microbes, which is a drawback. Despite the fact that NTS can provide a reference based on the detection data, physicians’ clinical expertise is ultimately what determines if the pathogen is a critical pathogen.

When we first evaluated the consistency of blood NTS and blood culture, we discovered that there was a 52.11% agreement between the two. The effectiveness of the two techniques in diagnosing clinical bloodstream infections was also contrasted. According to results of earlier studies (Wang et al., 2020a; Huang et al., 2021; Fu et al., 2022), it was discovered that the positive rate of blood NTS was significantly higher than that of blood culture (50.70%: 5.63%, *p* < 0.001), the sensitivity was also significantly higher than that of blood culture (62.50%: 7.14%, *p* < 0.001), and the NPV was also significantly higher than that of blood culture (40.00%: 22.39%). When we compare the overall outcomes of blood NTS and conventional pathogenic microbe identification (including blood culture, glucan test, galactomannan test, T cell spot test of tuberculosis infection, smear, etc.), the aforementioned conclusions are likewise drawn. One benefit of NTS is that it is almost unaffected to antibiotics, and the positive rate of culture changes significantly before and after antibiotic use (Cheng et al., 2019; Li et al., 2019). Culture detects living microorganisms, whereas NTS is based on microbial DNA detection. Some bacteria may be difficult to grow on conventional media, which may explain why NTS and culture are affected by antibiotics to varying degrees and also partially explains the difference in sensitivity between the two. This also suggests that NTS has a high false positive rate, which somewhat restricts its application, however its larger negative predictive value may be more useful. The findings of this study suggest that, particularly following empirical antibiotic treatment or the identification of specific pathogens, NTS has a greater diagnostic effectiveness than culture for clinical infections. Rapid detection is another benefit of NTS, with an initial report coming in 6 h after sample and a full report in 16 h (Wang et al., 2020a). In this investigation, 5 peripheral blood samples were collected from 5 individuals who took two NTS examinations. NTS can more quickly and precisely detect the alterations caused by infected strains in patients. When the No. 54 patients entered the intensive care unit, *Staphylococcus aureus* and *Acinetobacter baumannii* infections were found. NTS was negative after 3 days of therapy, and other indications including WBC, PCT, and others also indicated a decreased trend. When evaluating NTS, it was discovered that the infectious bacteria in the No. 37, No. 39, and No. 46 patients had changed, but the blood culture study findings did not reveal this change. This demonstrates that the use of NTS can reflect the clinical therapy impact more promptly. However, the pathogen load in the sample affects NTS detection. Wrong detection results may be produced when sample collection, processing, and storage are not uniform, there are technological faults, sample confusion, or insufficient initial sample concentration.

The main DDI organisms were Gram-negative bacteria, such as *Stenotrophomonas maltophilia*, *Pseudomonas aeruginosa*, methicillin-resistant *Staphylococcus aureus*, *Escherichia coli*, *Klebsiella pneumoniae*, and *Acinetobacter baumannii* (Bunsow et al., 2020). The presence of DDI in organ transplantation has a great impact on the prognosis of recipients. NTS was used in this investigation to identify 11 instances of *Escherichia coli* infection, 2 cases each of *Staphylococcus aureus* and *Pseudomonas aeruginosa*, and 1 case each of *Klebsiella pneumoniae*. However, one of the limitations of NTS was that it was unable to establish whether the identified bacteria were multidrug-resistant bacteria because it was unable to detect the drug sensitivity of the bacteria. However, NTS can preliminarily determine whether the bacteria are multidrug-resistant by comparing them to the published genome of multidrug-resistant bacteria. This can allow clinicians modify their usage of antibiotics and do target bacterial culture once more. Two NTS tests were performed on No. 69# patients on the same day, but only one result was positive, indicating some level of NTS error (van Dijk et al., 2018). Based on the patient’s clinical symptoms and other relevant investigations, doctors must evaluate the NTS results. In this study, it was discovered that NTS revealed that 12 recipients and their matching 7 donors both had the same pathogen infection, but culture did not reveal this phenomenon. This finding suggests that NTS may be more accurate than culture for predicting postoperative infection in recipients. These 12 recipients may have DDI, as defined by the term (Wolfe et al., 2019). Additionally, the infections were Gram-negative bacilli (*Escherichia coli*), which was consistent with earlier research (Ison et al., 2013). However, because we were unable to obtain the recipient’s pathogenic microorganisms’ test results before the organ transplant operation (no pathogenic microorganisms were tested prior to the operation) and the results of these pathogens’ drug sensitivity tests, it could not to definitively diagnose DDI, but may be NTS can be used as a powerful screening method and help to give timely treatment. In this study, the recipients included in the evaluation of the role of donor NTS results in predicting recipient infection were all renal recipients, which may be biased. In the future, a more thorough comparative analysis will be required to confirm this advantage.

This research contains some flaws. First off, the sample population in this study is limited and only originates from one center, which could have an impact on how well NTS can be detected. Second, all of the study subjects were deceased donors. Single patients made up the patient types, and they were all seriously ill. Therefore, it was not assessed if NTS might detect infections in other mild individuals. Thirdly, because this study was not retrospective cross-sectional, it was unable to determine how antibiotic therapy affected NTS outcomes. Fourthly, because the patients in this study had empirical anti-infective therapy before they were admitted to the intensive care unit, they might have some influence over the outcomes of the blood culture. Fifth, because this study only examined bacteria, fungus, mycoplasma, chlamydia, and other pathogens and did not compare viruses and spirochetes due to the lack of clinical case data, it was not feasible to assess the detection effectiveness of NTS for additional diseases. The NTS results were debated among the multidisciplinary group’s members, however there is no commonly agreed quantitative standard for the diagnosis of pathogenic microorganisms, thus there may be some bias due to possible false positives.

## 5. Conclusion

This study demonstrates the effectiveness of NTS for detecting possible bloodstream infections in deceased donors. It is not just an improvement over microbial culture methods; it also outperforms them. In terms of detection speed, positive rate, sensitivity, and identification of rare pathogens, it is superior than conventional microbiological detection. In addition, NTS may have an advantage in predicting recipient infection.

## Data availability statement

The datasets presented in this study can be found in online repositories. The names of the repository/repositories and accession number(s) can be found at: National Genomics Data Center - ‘CRA011898’ (https://www.cncb.ac.cn).

## Ethics statement

The studies involving humans were approved by the Renmin Hospital of Wuhan University’s Ethics Committee of Clinical Research. The studies were conducted in accordance with the local legislation and institutional requirements. Written informed consent for participation was not required from the participants or the participants’ legal guardians/next of kin in accordance with the national legislation and institutional requirements.

## Author contributions

ZY and YL: responsible for data collection, data statistics, data analysis, and paper writing. LZ, TQ, GL, ZC, XF, ZhoL, WW, and ZhaL: responsible for data collection. WX: responsible for data collection and research guidance. All authors contributed to the article and approved the submitted version.

## Conflict of interest

The authors declare that the research was conducted in the absence of any commercial or financial relationships that could be construed as a potential conflict of interest.

## Publisher’s note

All claims expressed in this article are solely those of the authors and do not necessarily represent those of their affiliated organizations, or those of the publisher, the editors and the reviewers. Any product that may be evaluated in this article, or claim that may be made by its manufacturer, is not guaranteed or endorsed by the publisher.
