# Supplementary material for: The utilization of nanopore targeted sequencing proves to be advantageous in the identification of infections present in deceased donors
Source: Front Microbiol. 2023 Aug 16;14:1238666. doi: 10.3389/fmicb.2023.1238666 (PMC10469296; doi:10.3389/fmicb.2023.1238666)
Supplement: Supplementary file 1 [file Table_1.DOC]

**Table S1 Detailed results of pathogens identified by Nanopore Target Sequencing(NTS) and Conventional microbiological testing**

| **Patient ID** | **Blood NTS results (reads)** | **Blood Culture results** | **Other Conventional microbiological testing results** | **Putative pathogens detected by Blood Culture** | **Putative pathogens detected by Blood NTS** |
| --- | --- | --- | --- | --- | --- |
| **NO.1#** | Negative | Negative | Negative | Negative | Negative |
| **NO.2#** | *Aspergillus penicillioides* (5936) | *Gram-positive bacillus, Paenibacillus* | Negative | Negative | *Aspergillus penicillioides* (5936) |
| **NO.3#** | Negative | Negative | Negative | Negative | Negative |
| **NO.4#** | *Pseudomonas aeruginosa* (191)*, Candida parapsilosis* (1582) | *Staphylococcus haemolyticus* | Negative | Negative | *Pseudomonas aeruginosa* (191)*, Candida parapsilosis* (1582) |
| **NO.5#** | Negative | *Staphylococcus Capitis, Staphylococcus hominis* | Negative | Negative | Negative |
| **NO.6#** | Negative | *Staphylococcus warneri* | Negative | Negative | Negative |
| **NO.7#** | *Aspergillus fumigatus*(2013) | Negative | Negative | Negative | *Aspergillus fumigatus*(2013) |
| **NO.8#** | Negative | *Staphylococcus cohnii* | Negative | Negative | Negative |
| **NO.9#** | Negative | Negative | Negative | Negative | Negative |
| **NO.10#** | *Acinetobacter lwoffii* (65) | Negative | Negative | Negative | *Acinetobacter lwoffii* (65) |
| **NO.11#** | *Corynebacterium spp*(308), *Staphylococcus saccharolyticus* (1653), *Gemella haemolysans* (123), *Abiotrophia defectiva* (144) | Negative | Negative | Negative | *Corynebacterium spp*(308) |
| **NO.12** | *Escherichia coli* (444) | Negative | Negative | Negative | *Escherichia coli* (444) |
| **NO.13#** | Negative | Negative | Negative | Negative | Negative |
| **NO.14#** | Negative | Negative | Negative | Negative | Negative |
| **NO.15#** | *Lactobacillus iners* (15214) | *Staphylococcus capitis, Coagulase negative staphylococcus* | Negative | Negative | Negative |
| **NO.16#** | *Enterobacter aerogenes* (146) | Negative | Negative | Negative | *Enterobacter aerogenes* (146) |
| **NO.17#** | Negative | Negative | Positive | Negative | Negative |
| **NO.18#** | *Staphylococcus aureus* (96), *Tsukamurella tyrosinosolvens* (86) | Negative | Negative | Negative | *Staphylococcus aureus (96)* |
| **NO.19#** | *Lactobacillus iners* (1154) | *Staphylococcus epidermidis* | Negative | Negative | Negative |
| **NO.20** | Negative | Negative | Negative | Negative | Negative |
| **NO.21#** | *Prevotella buccalis* (37) | *Staphylococcus cohnii* | Positive | Negative | Negative |
| **NO.22#** | Negative | Negative | Negative | Negative | Negative |
| **NO.23#** | *Enterococcus gallinarum* (218) | Negative | Negative | Negative | *Enterococcus gallinarum* (218) |
| **NO.24#** | *Achromobacter xylosoxidans* (1339),  *Hafnia alvei* (233) | Negative | Negative | Negative | Negative |
| **NO.25#** | Negative | *Staphylococcus epidermidis, Staphylococcus capitis* | Negative | Negative | Negative |
| **NO.26#** | Negative | Negative | Negative | Negative | Negative |
| **NO.27** | *Rothia mucilaginosa* (293), *Ralstonia pickettii* (221) | Negative | Negative | Negative | Negative |
| **NO.28#** | *Burkholderia gladioli* (105), *Sputum influenzae* (56) | Negative | Negative | Negative | *Burkholderia gladioli* (105), *Sputum influenzae* (56) |
| **NO.29#** | *Legionella spp* (363) | *Staphylococcus capitis* | Negative | Negative | *Legionella spp* (363) |
| **NO.30#** | Negative | Negative | Negative | Negative | Negative |
| **NO.31#** | *Finegoldia magna* (158),  *Comamonas terrigena* (40) | Negative | Negative | Negative | *Finegoldia magna* (158),  *Comamonas terrigena* (40) |
| **NO.32#** | *Escherichia coli* (492),  *Burkholderia vietnamiensis* (73) | *Klebsiella pneumoniae* | Positive | *Klebsiella pneumoniae* | *Escherichia coli* (492),  *Burkholderia vietnamiensis* (73) |
| **NO.33#** | *Moraxella osloensis* (1616), *Staphylococcus saprophyticus* (1253) | Negative | Positive | Negative | Negative |
| **NO.34#** | *Escherichia coli* (434),  *Corynebacterium jeikeium* (135) | Negative | Negative | Negative | *Escherichia coli* (434) |
| **NO.35#** | *Escherichia cloacae* (4176),  *Candida albicans* (492) | *Staphylococcus epidermidis* | Negative | Negative | *Escherichia cloacae* (4176),  *Candida albicans* (492) |
| **NO.36#** | *Pseudomonas fluorescens* (209),  *Escherichia coli* (59) | Negative | Positive | Negative | *Pseudomonas fluorescens* (209),  *Escherichia coli* (59) |
| **NO.37#** | *Escherichia coli* (572), *Acinetobacter guillouiae* (293), *Pseudomonas stutzeri* (116), *Corynebacterium mucifaciens* (4948) | Negative | Negative | Negative | *Escherichia coli* (973), *Acinetobacter guillouiae* (293), *Pseudomonas stutzeri* (116), *Corynebacterium mucifaciens* (4948) |
| **NO.38#** | *Pseudomonas aeruginosa* (40), *Leptotrichia wadei* (144) | *Staphylococcus equorum* | Negative | Negative | *Pseudomonas aeruginosa* (40) |
| **NO.39#** | *Escherichia coli* (256),  *Anaerococcus octavius* (1481),  *Acinetobacter haemolyticus* (218) | Negative | Negative | Negative | *Escherichia coli* (256),  *Anaerococcus octavius* (1481),  *Acinetobacter haemolyticus* (218) |
| **NO.40#** | *Escherichia coli* (1120), *Corynebacterium jeikeium* (82)，  *Pseudomonas stutzeri* (416)，*Acinetobacter baumannii*(117),  *Enterococcus faecalis* (230),  *Candida tropicalis* (14123) | *Candida tropicalis, Enterococcus faecalis* | Positive | *Candida tropicalis, Enterococcus faecalis* | *Escherichia coli* (1120),  *Pseudomonas stutzeri* (416)，*Acinetobacter baumannii*(117),  *Enterococcus faecalis* (230), *Candida tropicalis* (14123) |
| **NO.41#** | *Burkholderia cepacia* (1180) | Negative | Negative | Negative | *Burkholderia cepacia* (1180) |
| **NO.42#** | *Pseudomonas stutzeri* (510) | Negative | Positive | Negative | *Pseudomonas stutzeri* (510) |
| **NO.43#** | *Staphylococcus epidermidis* (457) | Negative | Negative | Negative | Negative |
| **NO.44** | *Corynebacterium* *kroppenstedtii* (327) | Negative | Negative | Negative | Negative |
| **NO.45#** | *Acidovorax delafieldii* (3463) | Negative | Positive | Negative | Negative |
| **NO.46** | *Ralstonia pickettii* (3180) | *Staphylococcus epidermidis* | Negative | Negative | Negative |
| **NO.47#** | Negative | Negative | Positive | Negative | Negative |
| **NO.48#** | Negative | Negative | Negative | Negative | Negative |
| **NO.49#** | *Enterobacter cloacae* (36), *Citrobacter freundii* (218) | Negative | Negative | Negative | *Enterobacter cloacae* (36) |
| **NO.50#** | *Escherichia coli* (548), *Pseudomonas hibiscicola* (203) | Negative | Negative | Negative | *Escherichia coli* (548), *Pseudomonas hibiscicola* (203) |
| **NO.51#** | *Escherichia coli* (156), *Moraxella osloensis* (376) | Negative | Negative | Negative | *Escherichia coli* (156) |
| **NO.52#** | *Enterobacter cloacae* (35), *Salmonella enterica* (386), *Pseudomonas oryzihabitans* (81) | Negative | Negative | Negative | *Enterobacter cloacae* (35), *Salmonella enterica* (386), *Pseudomonas oryzihabitans* (81) |
| **NO.53#** | Negative | Negative | Negative | Negative | Negative |
| **NO.54#** | *Staphylococcus aureus* (978), *Acinetobacter baumannii* (676), *Yarrowia lipolytica* (235) | Negative | Negative | Negative | *Staphylococcus aureus* (978), *Acinetobacter baumannii* (676) |
| **NO.55** | Negative | Negative | Positive | Negative | Negative |
| **NO.56#** | *Gordonia bronchialis* (475) | Negative | Negative | Negative | *Gordonia bronchialis* (475) |
| **NO.57#** | *Moraxella osloensis* (455) | *Acinetobacter baumannii* | Positive | *Acinetobacter baumannii* | Negative |
| **NO.58#** | *Escherichia coli* (914), *Leuconostoc citreum* (820) | Negative | Negative | Negative | *Escherichia coli* (914) |
| **NO.59#** | *Klebsiella pneumoniae* (515), *Lactobacillus iners* (11145) | Negative | Negative | Negative | *Klebsiella pneumoniae* (515) |
| **NO.60#** | Negative | Negative | Negative | Negative | Negative |
| **NO.61#** | *Gordonia bronchialis* (21), *Ralstonia pickettii* (221) | Negative | Negative | Negative | *Gordonia bronchialis* (21) |
| **NO.62#** | *Parvimonas micra* (259), *Enterococcus faecalis* (23) | Negative | Negative | Negative | *Parvimonas micra* (259), *Enterococcus faecalis* (23) |
| **NO.63#** | *Paracoccus marinus* (496), *Bordetella petrii* (234) | Negative | Negative | Negative | Negative |
| **NO.64#** | *Fusarium spp.* (810), *Bergeyella porcorum* (161) | Negative | Negative | Negative | *Fusarium spp.* (810) |
| **NO.65#** | P*aracoccus yeei* (246) | Negative | Positive | Negative | Negative |
| **NO.66#** | *Lactobacillus iners* (346) | *Staphylococcus haemolyticus* | Positive | Negative | Negative |
| **NO.67#** | *Burkholderia spp.* (17927) | Negative | Positive | Negative | *Burkholderia spp.* (17927) |
| **NO.68#** | *Klebsiella variicola* (177), *Diphthemid bacillus* (171), *Staphylococcus saprophyticus* (1349) | *Klebsiella pneumoniae* | Positive | *Klebsiella pneumoniae* | *Klebsiella variicola* (177), *Diphthemid bacillus* (171) |
| **NO.69#** | *Ochrobactrum anthropic* (1338) | Negative | Positive | Negative | Negative |
| **NO.70#** | *Escherichia coli* (271), *Anaerobicbacteria octavius* (167), *Candida parapsilosis* (12486), *Cladosporium sp.* (1122) | Negative | Negative | Negative | *Escherichia coli* (271), *Anaerobicbacteria octavius* (167), *Candida parapsilosis* (12486) |
| **NO.71#** | *Staphylococcus saccharolyticus* (1075) | Negative | Positive | Negative | Negative |

#: Infection patient.

**Table S2 Performance of Blood Nanopore Target Sequencing (NTS) and Conventional microbiological testing in the diagnosis of clinical infectious**

|  |  | **Infection(56)** | **Non-infection(15)** | **Sensitivity%(95%CI)** | **Specificity%(95%CI)** | **PPV%(95%CI)** | **NPV%(95%CI)** |
| --- | --- | --- | --- | --- | --- | --- | --- |
| **Blood NTS** | + | 35 | 1 | 62.50(48.52, 74.77) | 93.33(66.03, 99.65) | 97.22(83.80, 99.85) | 40.00(24.35, 57.79) |
|  | - | 21 | 14 |  |  |  |  |
| **Conventional**  **microbiological**  **testing** | + | 15 | 2 | 26.79(16.23, 40.54) | 86.67(58.39, 97.66) | 88.24(62.25, 97.94) | 24.07(13.92, 37.94) |
|  | - | 41 | 13 |  |  |  |  |

PPV=Positive Predictive Value, NPV=Negative Predictive Value.

**Table S3** The results of pathogenic microorganism examination of deceased donors and their corresponding renal recipients

| **Deceased donors** | | | **Renal recipients** | | | | | |
| --- | --- | --- | --- | --- | --- | --- | --- | --- |
| **Patient ID** | **Blood NTS results (reads)** | **Blood Culture results** | **Patient ID** | **Blood Culture results** | **Urine culture** | **Blood NTS results (reads)** | **Urine NTS results (reads)** | **Surgical site secretion culture results** |
| **NO.3** | Negative | Negative | **NO.5** | *Enterococcus faecium* | *Enterococcus faecium*, *proteus mirabilis* | *Finegoldia magna* (277) | Negative | Negative |
|  |  |  | **NO.6** | Negative | Negative | non-implementation | *Enterococcus avium* (26) | Negative |
| **NO.9** | Negative | Negative | **NO.16** | *Candida glabrata* | Negative | Negative | non-implementation | non-implementation |
| **NO.12** | *Escherichia coli* (444) | Negative | **NO.19** | Negative | *Candida parapsilosis* | non-implementation | non-implementation | Negative |
| **NO.14** | Negative | Negative | **NO.21** | *Enterococcus faecium* | Negative | Negative | non-implementation | Negative |
|  |  |  | **NO.22** | Negative | Negative | non-implementation | non-implementation | *Klebsiella pneumoniae* |
| **NO.28** | *Burkholderia gladioli* (105),  *Sputum influenzae* (56) | Negative | **NO.47** | Negative | Negative | non-implementation | *Stenotrophomonas maltophilia* (96) | Negative |
|  |  |  | **NO.48** | Negative | Negative | non-implementation | *Acinetobacter johnsonii* (6) | non-implementation |
| **NO.29** | *Legionella spp* (363) | Negative | **NO.50** | Negative | *Candida parapsilosis* | *Stretpococcus mitis* (166) | Negative | Negative |
| **NO.30** | Negative | Negative | **NO.51** | Negative | Negative | non-implementation | *Rickettsia-burnetii* (12) | Negative |
| **NO.32** | *Escherichia coli* (492),  *Burkholderia vietnamiensis* (73) | Negative | **NO.55** | Negative | Negative | *Escherichia coli* (2395) | non-implementation | Negative |
|  |  |  | **NO.56** | Negative | Negative | *Escherichia coli* (5151), *Pseudomonas stutzeri* (72) | non-implementation | *Escherichia coli* |
| **NO.33** | Negative | Negative | **NO.57** | Negative | Negative | *Escherichia coli* (405), *Pseudomonas luteola* (388), *Comamonas testosteroni* (1545), *Candida parapsilosis*(1285) | Negative | Negative |
|  |  |  | **NO.58** | Negative | Negative | *Escherichia coli* (1555) | *Escherichia coli* (117) | Negative |
| **NO.34** | *Escherichia coli* (434) | Negative | **NO.59** | Negative | Negative | *Escherichia coli* (324), *Burkholderia cepacia* (1277), *Acinetobacter johnsonii* (4434) | Negative | Negative |
|  |  |  | **NO.60** | Negative | Negative | *Escherichia coli* (89), *Streptococcus oralis* (111) | Negative | Negative |
| **NO.36** | *Pseudomonas fluorescens* (209),  *Escherichia coli* (59) | Negative | **NO.62** | Negative | Negative | *Pseudomonas aeruginosa* (127), *Escherichia coli* (4227) | *Enterococcus gallinarum* (16) | Negative |
|  |  |  | **NO.63** | Negative | Negative | *Staphylococcus aureus* (664), *Escherichia coli* (1297)，*Pseudomonas stutzeri* (228) | Negative | Negative |
| **NO.37** | *Escherichia coli* (973), *Acinetobacter guillouiae* (293), *Pseudomonas stutzeri* (116), *Corynebacterium mucifaciens* (4948) | Negative | **NO.64** | Negative | Negative | *Escherichia coli* (2352) | non-implementation | non-implementation |
| **NO.38** | *Pseudomonas aeruginosa* (40) | Negative | **NO.66** | Negative | Negative | *Escherichia coli* (910) | non-implementation | Negative |
| **NO.39** | *Escherichia coli* (256),  *Anaerococcus octavius* (1481),  *Acinetobacter haemolyticus* (218) | Negative | **NO.68** | Negative | Negative | *Escherichia coli* (132), *Pseudomonas luteola* (309) | Negative | non-implementation |
|  |  |  | **NO.69** | Negative | Negative | *Escherichia coli* (134), *Streptococcus oralis* (293), *Pseudomonas stutzeri* (270), *Malassezia.restricta* (361) | Negative | Negative |
| **NO.43** | Negative | Negative | **NO.74** | Negative | Negative | *Serratia marcescens* (421) | non-implementation | Negative |
| **NO.45** | Negative | Negative | **NO.79** | Negative | Negative | *Fusarium solani* (60) | non-implementation | Negative |
| **NO.46** | Negative | Negative | **NO.80** | Negative | Negative | *Acinetobacter radioresistens* (543), *Acinetobacter baumannii* (156), *Escherichia coli* (22), *Candida albicans* (168) | *Acinetobacter baumannii* (1111), *Klebsiella pneumoniae* (324), *Pseudomonas aeruginosa* (225) | Negative |
| **NO.47** | Negative | Negative | **NO.82** | Negative | Negative | *Escherichia coli* (215), *Pseudomonas aeruginosa* (23) | non-implementation | Negative |
| **NO.48** | Negative | Negative | **NO.84** | non-implementation | Negative | *Pseudomonas aeruginosa* (369), *Escherichia coli* (296) | non-implementation | Negative |
|  |  |  | **NO.85** | non-implementation | Negative | *Acinetobacter junii* (643), *Pseudomonas luteola* (212) | non-implementation | Negative |
| **NO.49** | *Enterobacter cloacae* (36) | Negative | **NO.86** | non-implementation | Negative | Negative | *Candida albicans* (81) | Negative |
|  |  |  | **NO.87** | non-implementation | Negative | *Escherichia coli* (53) | *Candida albicans* (145) | Negative |
| **NO.50** | *Escherichia coli* (548), *Pseudomonas hibiscicola* (203) | Negative | **NO.88** | non-implementation | Negative | *Pseudomonas stutzeri* (78), *Escherichia coli* (20) | non-implementation | Negative |
|  |  |  | **NO.89** | non-implementation | Negative | *Pseudomonas stutzeri* (364), *Escherichia coli* (169), *Pseudomonas oryzihabitans* (250) | non-implementation | Negative |
| **NO.51** | *Escherichia coli* (156) | Negative | **NO.90** | Negative | Negative | *Escherichia coli* (67) | non-implementation | Negative |
|  |  |  | **NO.91** | Negative | Negative | *Pseudomonas stutzeri* (100) | non-implementation | Negative |
| **NO.52** | *Enterobacter cloacae* (35), *Salmonella enterica* (386), *Pseudomonas oryzihabitans* (81) | Negative | **NO.92** | non-implementation | non-implementation | *Staphylococcus aureus* (100), *Escherichia coli* (21) | Negative | non-implementation |
|  |  |  | **NO.93** | non-implementation | non-implementation | *Staphylococcus aureus* (597), *Pseudomonas luteola* (149) | Negative | non-implementation |
| **NO.53** | Negative | Negative | **NO.94** | non-implementation | Negative | *Enterococcus faecium* (1760)，*Escherichia coli* (731) | Negative | non-implementation |
|  |  |  | **NO.95** | non-implementation | Negative | *Pseudomonas stutzeri* (711), *Burkholderia cepacia* (244), *Enterobacter cloacae* (468) | Negative | Negative |
| **NO.54** | *Staphylococcus aureus* (978), *Acinetobacter baumannii* (676) | Negative | **NO.96** | Negative | Negative | *Escherichia coli* (349), *Pseudomonas aeruginosa* (52) | non-implementation | Negative |
|  |  |  | **NO.97** | Negative | Negative | *Pseudomonas stutzeri* (346) | non-implementation | Negative |
| **NO.55** | Negative | Negative | **NO.98** | non-implementation | Negative | *Escherichia coli* (2042) | non-implementation | Negative |
| **NO.56** | *Gordonia bronchialis* (475) | Negative | **NO.10** | Negative | Negative | *Candida albicans* (2881) | non-implementation | Negative |
|  |  |  | **NO101** | Negative | Negative | *Pseudomonas stutzeri* (2907) | non-implementation | Negative |
| **NO.57** | Negative | *Acinetobacter baumannii* | **NO.103** | Negative | Negative | *Pseudomonas stutzeri* (158) | non-implementation | Negative |
| **NO.58** | *Escherichia coli* (914) | Negative | **NO.105** | Negative | Negative | *Enterococcus casseliflavus* (239) | non-implementation | Negative |
| **NO.64** | *Fusarium spp.* (810) | Negative | **NO.116** | Negative | *Enterococcus faecium* | non-implementation | Negative | Negative |
| **NO.67** | *Burkholderia spp.* (17927) | Negative | **NO.122** | Negative | Negative | non-implementation | *Candida parapsilosis* (1049) | Negative |

NTS= Nanopore Target Sequencing
